# Lumbar spine segmentation method based on deep learning

**DOI:** 10.1002/acm2.13996

**Published:** 2023-04-20

**Authors:** Hongjiang Lu, Mingying Li, Kun Yu, Yingjiao Zhang, Liang Yu

**Affiliations:** ^1^ Department of Radiology 903 Hospital of the Joint Service Support Force of the Chinese People's Liberation Army Hangzhou Zhejiang China; ^2^ Department of Head and Neck & Thyroid Surgery Zhejiang Provincial People's Hospital, Affiliated People's Hospital, Hangzhou Medical College Hangzhou Zhejiang China; ^3^ Department of Gastroenterology 903 Hospital of the Joint Service Support Force of the Chinese People's Liberation Army Hangzhou Zhejiang China; ^4^ Department of Radiology Zhejiang Provincial People's Hospital, Affiliated People's Hospital, Hangzhou Medical College Hangzhou Zhejiang China

**Keywords:** CT images, lumbar vertebrae segmentation, spinal anomalies, surgical treatment

## Abstract

Aiming at the difficulties of lumbar vertebrae segmentation in computed tomography (CT) images, we propose an automatic lumbar vertebrae segmentation method based on deep learning. The method mainly includes two parts: lumbar vertebra positioning and lumbar vertebrae segmentation. First of all, we propose a lumbar spine localization network of Unet network, which can directly locate the lumbar spine part in the image. Then, we propose a three‐dimensional XUnet lumbar vertebrae segmentation method to achieve automatic lumbar vertebrae segmentation. The method proposed in this paper was validated on the lumbar spine CT images on the public dataset VerSe 2020 and our hospital dataset. Through qualitative comparison and quantitative analysis, the experimental results show that the method proposed in this paper can obtain good lumbar vertebrae segmentation performance, which can be further applied to detection of spinal anomalies and surgical treatment.

## 1 INTRODUCTION

Over the past few decades, various medical imaging modalities, including x‐rays, computed tomography (CT), magnetic resonance imaging (MRI), and positron emission tomography (PET) have been widely used to examine spinal structural abnormalities.^1^, ^2^ CT imaging technology has been used to intuitively observe the internal information of the human body and count the volume information of various tissues due to its advantages of fast scanning speed and clear image generation.^3^ Accurate lumbar vertebrae segmentation is an important step in subsequent analysis and treatment, including examining abnormal vertebrae fractures, biomechanical modeling, lumbar inter‐body fusion, and anterior and posterior lumbar inter‐body fusion.^4^, ^5^ In particular, these analyses and treatments place high demands on the accuracy of lumbar inter‐body segmentation. Before the maturity of artificial intelligence technology, the segmentation of vertebral bodies was mostly done manually by multiple experienced radiologists. However, the artificial vertebrae delineation method is labor‐intensive and time‐consuming, and the delineation standards of different radiologists are inconsistent, resulting in large differences in the segmentation results of the vertebrae. Therefore, an automatic and efficient automatic segmentation method of vertebrae is of great significance for the subsequent morphological analysis and clinical treatment.

The essence of automatic segmentation of vertebral bodies from CT images is to segment the vertebraes in CT images to provide a reliable basis for clinical diagnosis and treatment research. It can be understood as a pixel‐level classification method. Traditional image segmentation methods such as threshold‐based and region‐based segmentation can be applied to medical image segmentation. However, due to the influence of imaging equipment, imaging principles and other factors, the content and shape of medical images are relatively complex, and these traditional methods still have great challenges in segmentation accuracy.

Recently, significant progress has been made in image segmentation technology based on deep learning mainly with convolutional neural networks. There are many segmentation architectures with good performance, such as fully convolutional neural (FCN),^6^ SegNet,^7^ U‐Net,^8^ and 3D UNet,^9^ etc. The networks used in many image segmentation studies are based on the above networks or their corresponding 3D versions. The U‐Net^8^ model adopts a symmetrical U‐shaped structure and achieves good results in many medical segmentation tasks, but its skip‐layer connection forces the encoder and decoder to perform feature fusion only on the same depth layer. Neglecting the fusion of semantic information with different scales will result in the loss of certain details. UNet++^10^ optimizes according to the structure of the UNet model, alleviates the unknown network depth with an efficient ensemble of U‐Nets of different depths, redesigns skip connections to aggregate features of different semantic scales at the decoder sub‐networks, and designs a pruning scheme to accelerate the inference speed of UNet++.^10^ UNet3+^11^ utilizes full‐scale skip connections and deep supervisions. DeepLab V1^12^ uses VGG16^13^ as the Backbone, and uses atrous convolution and conditional random fields to improve the classification accuracy. DeepLab V2^14^ uses ResNet‐101^15^ as Backbone, uses atrous convolution more flexibly, and proposes atrous space pyramid pooling. DeepLab V3^16^ abandons conditional random fields, improves atrous space pyramid pooling and uses atrous convolution to deepen the network.

Aiming at the difficulties of lumbar vertebrae segmentation in CT images, we propose a two‐stage automatic lumbar vertebrae segmentation method based on deep learning. The proposed method mainly includes two stages of lumbar vertebra positioning and lumbar vertebrae segmentation. First of all, we propose a lumbar spine localization network of Unet network, which can directly locate the lumbar spine part in the image. Then, this paper proposes a three‐dimensional XUnet lumbar vertebrae segmentation method to achieve automatic lumbar vertebrae segmentation. Different from existing methods, a novel low‐parametric nature of Inception block is introduced to capture the rich feature information in the encoder part and multi‐scale features are fused in the decode part to taking into account that the surrounding context to enable sound segmentation performance. The method proposed in this paper was validated on the lumbar spine CT images on the public dataset VerSe 2020 and our hospital dataset.^17^ Through qualitative comparison and quantitative analysis, the experimental results show that the method proposed in this paper can obtain good lumbar vertebrae segmentation performance, which can be further applied to detection of spinal anomalies and surgical treatment.

## 2 RELATED WORKS

In recent years, various machine learning algorithms have also been widely used in the field of image segmentation. Jimenez‐Pastor et al.^18^ introduced a two‐stage decision forest and morphological image processing technique to automatically detect and identify vertebral bodies in arbitrary field‐of‐view volume CT scans. Combining the convolutional neural network with the deformation model, Korez et al.^19^ learn the spine features and output the probability map of the spine through the convolutional neural network, so as to guide the deformation model to generate the boundary of the spine, and finally realize the spine segmentation.

Benefiting from the improvement of computational resource performance and the increase of datasets available for training, deep learning is widely used in image segmentation. Model‐based spine segmentation methods are gradually being replaced by data‐driven deep learning methods as public spine image datasets become more sophisticated. Sekuboyina et al.^20^ used two convolutional neural networks for spine localization and segmentation, respectively. The input of the localization network is a two‐dimensional slice of the spine image, and the ground‐truth of the localization network is obtained by downsampling the real spine mask, using patches to segment the vertebrae one by one. Similarly, Janssens et al.^21^ used two three‐dimensional FCN^6^ networks for spine localization and segmentation. The localization network uses regression to obtain the bounding box of the spine to localize the spine. The segmentation network directly performs voxel‐level multi‐classification. Lessmann et al.^22^ used only one three‐dimensional FCN^6^ for spine segmentation. They use an iterative method to segment the vertebrae one by one according to the prior rules of the appearance of vertebrae, and determine whether the vertebrae in the current block have been segmented by adding a memory component. In addition, there is a classification component dedicated to labeling vertebrae that have been segmented. However, the above‐mentioned method for segmenting the spine using blocks is difficult to solve the problem of overlapping between the vertebrae. Paye et al.^23^ proposed a multi‐stage method for spine segmentation, first using 3D U‐Net to roughly localize the spine, then using SpatialConfiguration‐Net^24^ to perform heat map regression for spine localization and identification, and finally using 3D U‐Net to perform binary segmentation on the identified vertebrae. The above deep learning‐based spine segmentation research has achieved good segmentation results.

## 3 METHOD

### 3.1 Method overview

Aiming at the difficulties of lumbar vertebrae segmentation in CT images, we propose a novel two‐stage lumbar vertebrae segmentation method based on deep learning. The method mainly includes two stages of lumbar vertebra positioning and lumbar vertebrae segmentation. First of all, we propose a lumbar spine localization network of Unet network, which can directly locate the lumbar spine part in the image, and crop out the image containing the lumbar spine, so as to improve the performance of subsequent segmentation. Then, we propose a three‐dimensional XUnet lumbar vertebrae segmentation method with an encoder‐decoder framework to achieve automatic lumbar vertebrae segmentation. At the encoder part, a novel low‐parametric nature of Inception block is introduced to capture the rich feature information. At the decoder part, the multi‐scale features are fused in the decode part to taking into account that the surrounding context to enable sound segmentation performance.

### 3.2 Spine positioning method and image cropping

#### 3.2.1 UNet—based localization network model

**FIGURE 1:**
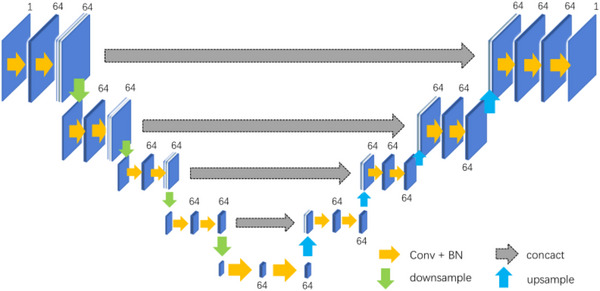
UNet‐based localization network.

In order to obtain the accurate positioning of the lumbar spine, this paper proposes a positioning network model based on UNet, as shown in Figure 1. The network model structure is as follows:

1. Suppose the input is $X\in R^{H*W*D*C}$, and $X_{i}\;\in \;R^{H_{i}*W_{i}*D_{i}*C_{i}},i\in$[1, 4] is obtained after feature extraction by the encoder, then the decoding feature $Y_{i}$ can be expressed as:
(1) $$\begin{array}{c} \begin{array}{c} Y_{i}=\sigma \left(f_{3*3*3}\left(Concat\left\{X_{i}\left[16:48\right],upsample\left(X_{i+1}\right)\right\}\right)\right) \end{array} \end{array}$$

where downsampl is downsampling, upsample is upsampling, σ is the activation function, $f_{3*3*3}$means that the convolution operation is performed with a convolution kernel of 3*3*3 size.

1. According to the method described in Equation (1), multi‐scale feature fusion is realized in the decoding process. The specific flow of data in the network is as follows:

The first layer of the encoder: the input scale is 96*96*128*1, and the feature map $X_{1}\;$is obtained after two convolutions to extract the feature, and the feature map $X_{1}^{\prime }$ with 64 channels and the size of 48*48*64 is obtained after one down sampling;

The second layer of the encoder: the input feature map $X_{1}{}^{\prime }$ with a scale of 48*48*64, the feature map *X*_2_ is obtained after two convolutions to extract the feature, and the feature map $X_{2}{}^{\prime }$ with 64 channels and a size of 24*24*32 is obtained after one down sampling;

The third layer of the encoder: the input feature map $X_{2}{}^{\prime }$ with a scale of 24*24*32, the feature map *X*_3_ is obtained after two convolutions to extract the feature, and the feature map $X_{3}{}^{\prime }$ with 64 channels and size 12*12*16 is obtained after one down sampling;

The fourth layer of the encoder: the input feature map $X_{3}{}^{\prime }$ with a scale of 12*12*16, the feature map *X*_4_ is obtained after two convolutions to extract the feature, and the feature map $X_{4}{}^{\prime }$ with 64 channels and size 6*6*8 is obtained after one down sampling;

The fifth layer of the encoder: the input feature map $X_{4}{}^{\prime }$ with a scale of 6*6*8, and the feature map $X_{5}\;$with 64 channels and a size of 6*6*8 is obtained after two convolutions to extract the feature;

The first layer of the decoder: the multi‐scale features *X*_4_[16:48] and the deconvolutional feature map *X*_5_ are spliced along the channel direction to obtain features *Y*_1_, and then 64 channels with a size of 12*12* 16 feature maps $Y_{1}{}^{\prime }$ is obtained by performing feature fusion through two convolutions;

In the second layer of the decoder, the multi‐scale feature *X*_3_[16:48] and the up‐sampled feature map $Y_{1}^{{}^{\prime }}$ are spliced along the channel direction to obtain features *Y*_2_, and then 64 channels with a size of 24*24*32 feature map $Y_{2}{}^{\prime }$ is obtained by performing feature fusion through two convolutions;

In the third layer of the decoder, the multi‐scale feature *X*_2_[16:48] and the up‐sampled feature map $Y_{2}^{{}^{\prime }}$are spliced along the channel direction to obtain features *Y*_3_, and then 64 channels with a size of 48*48*64 feature map $Y_{3}{}^{\prime }$ is obtained by performing feature fusion by twice using convolutional layer; and

In the fourth layer of the decoder, the multi‐scale feature *X*_1_[16:48] and the upsampled feature map $Y_{3}{}^{\prime }$ are spliced along the channel direction to obtain the feature map *Y*_4_, and then 64 channels, with a size of 96*96*128 feature map $Y_{4}{}^{\prime }$ is obtained by performing feature fusion through two convolutions. Finally, a convolution kernel of size 1*1*1 is used to generate a localization heat map.

Through the method proposed in this paper, the precise positioning of the lumbar vertebrae can be obtained, which assists the subsequent accurate segmentation of the lumbar vertebrae.

#### 3.2.2 Image cropping

After completing the localization of the lumbar vertebrae, a bounding box containing the lumbar vertebrae can be obtained, and then the image containing the lumbar vertebrae can be cropped. The calculation of the bounding box requires the first and last occurrences of subscripts with a HU value greater than a certain threshold in each dimension of the calculation site. This pair of subscripts constitutes the range of the bounding box in the corresponding dimension. After obtaining the three‐dimensional range, it can be directly cropped, but the model prediction result may have multiple concentrated bright areas, the boundary range obtained by using the above algorithm will be too large, and there will be many black areas in the cropped image. Therefore, it is also necessary to determine the area with the largest range of light. In addition, in order to ensure that the cropped image completely contains the lumbar spine, the calculated bounding box needs to be expanded. For example, each dimension is expanded by 0.1 times the corresponding size of the image. Crop the image according to the resulting bounding box.

**FIGURE 2:**
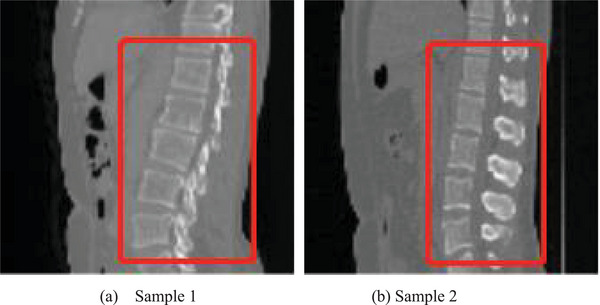
Cropping result of the lumbar vertebrae image for two samples, where the lumbar vertebrae region is in the rectangle with red line.

This scheme uses four neighborhoods to obtain the range of the lumbar vertebra to be selected, and selects the voxels whose HU value is greater than 0.3 times the maximum HU value of the image as the possible lumbar vertebrae, and finally expands the obtained bounding box in each dimension by 0.1 times the size of the corresponding image. Figure 2 shows the cropping result of the lumbar vertebrae image for two samples, where the lumbar vertebrae region is in the rectangle with red line. It can be seen that a lot of redundant information is deleted after cropping.

### 3.3 A lumbar spine segmentation model based on 3D multi‐scale XUNet

**FIGURE 3:**
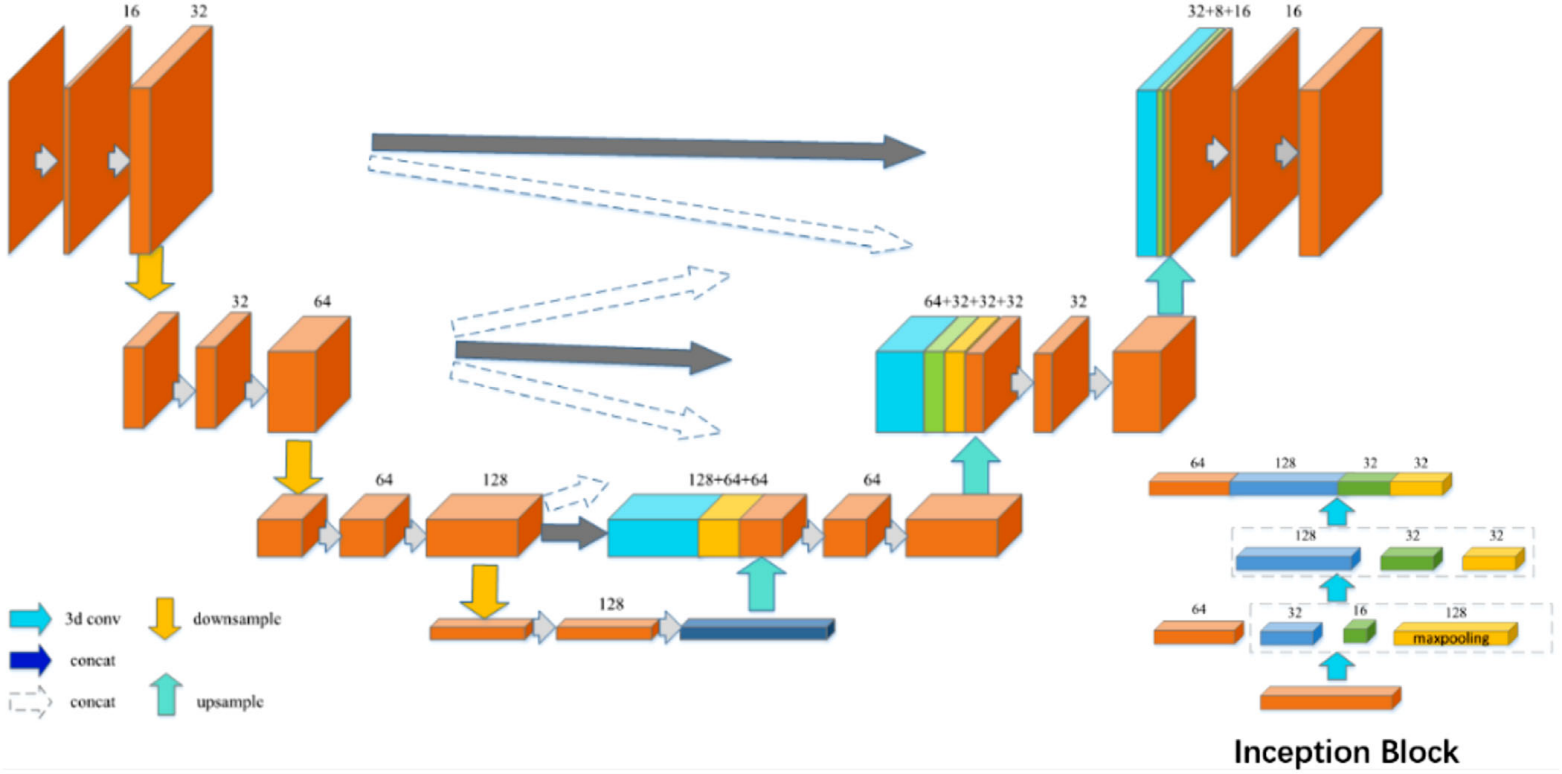
Schematic diagram of 3D multi‐scale XUNet network.

In order to accurately segment the lumbar spine, we propose a 3D multi‐scale XUNet‐based spine segmentation method (Figure 3). XUnet uses the Inception block for feature extraction, where the Inception block includes two feature extraction layers, the first layer includes three convolution blocks (denoted as convblock1‐1, convblock1‐2, and convblock1‐3) and a maximum pooling, where convblock1‐1 consists of 64 convolution kernels of size 1*1 *1, convblock1‐2 consists of 32 convolution kernels of size 1*1 *1, convblock1‐3 consists of 16 convolution kernels of size 3*3*3 convolution kernels. The second layer includes three convolution blocks (denoted as convblock2‐1, convblock2‐2, and convblock2‐3), where convblock2‐1 consists of 128 convolution kernels of size 3*3*3, and convblock2‐2 consists of 32 It consists of 5*5*5 convolution kernels, and convblock2‐3 consists of 32 1*1*1 convolution kernels. The feature extraction results from convblock1‐1, convblock2‐1, convblock2‐2, and convblock2‐3 are concatenated and fused as the final output of the Inception block. This enables the aggregation of features at different semantic scales. At the same time, the Inception block is used to replace the convolution operation, which further reduces the redundant parameters in the network while deepening the width and depth of the network and improving the expressive ability of the network.

The specific structure of the network is as follows:

1. Suppose the input is $X\in R^{H*W*D*C}$,$X_{i}\;\in \;R^{H_{i}*W_{i}*D_{i}*C_{i}},i\in \left[1,4\right]$ is obtained after feature extraction by the encoder, then the decoding feature $Y_{i}\;$can be expressed as:
(2) $$\begin{array}{ccc} Yi & = & \sigma \left(f_{3*3*3}(Concat\{downsample\left(X_{i-1}\right),\right.\\ & & \left.X_{i},\;upsample\left(X_{i+1}\right),\;upsample\left(Y_{i+1}\right)\})\right) \end{array}$$

where downsampl is downsampling, upsample is upsampling, and σ is an activation function, $f_{3*3*3}$means that the convolution operation is performed with a convolution kernel of 3 *3*3 size.

1. Multi‐scale feature fusion is realized in the decoding process, and the specific flow of data in the network is as follows:

The first layer of the encoder: the input scale is 64*64*128*1, the feature map *X*_1_ is obtained after two convolutions to extract the feature, and the feature map with 32 channels and the size of 32*32*64 is obtained after one downsampling;

The second layer of the encoder: the input feature map $X_{1}{}^{\prime }$ with a scale of 32*32*64, the feature map is obtained after two convolutions to extract the feature, and the feature map $X_{2}{}^{\prime }$ with 64 channels and a size of 16* 16 *32 is obtained after one downsampling;

The third layer of the encoder: input feature map *X*_3_ with a scale of 16 $X_{2}{}^{\prime }$*16*32, extract features through two convolutions to obtain a feature map, and a feature map $X_{3}{}^{\prime }$ with 128 channels and a size of 8*8*16 is obtained after one downsampling;

The fourth layer of the encoder: the input feature map $X_{3}{}^{\prime }$ with a scale of 8*8*16, after a convolution and an Inception block to extract features, and a feature map *X*_4_ with 256 channels and a size of 4*4*8 is obtained;

The first layer of the decoder: the multi‐scale feature maps *X*_2_, *X*_3_ and the deconvolutional feature map *X*_4_ are spliced along the channel direction to obtain the feature map *Y*_1_, and then feature map $Y_{1}{}^{\prime }$ with 128 channels and a size of 16*16*32 is obtained by performing feature fusion through two convolutions;

In the second layer of the decoder, the multi‐scale feature maps $X_{1},\;\;X_{2},\;\;X_{3}$ and the up‐sampled feature map $Y_{1}^{{}^{\prime }}$are spliced along the channel direction to obtain feature map *Y*_2_, and then feature map $Y_{2}{}^{\prime }$ with 64 channels and a size of 32*32*64 is obtained by performing feature fusion through two convolutions; and

In the third layer of the decoder, the feature maps *X*_1_, *X*_2_ and the multi‐scale feature map $Y_{2}{}^{\prime }$ are spliced along the channel direction to obtain the feature map *Y*_3_, and then a feature map $Y_{3}{}^{\prime }$ with 32 channels and a size of 64*64*128 is obtained by performing feature fusion through two convolutions. Finally, combine the Softmax function to complete the segmentation and return the segmentation result graph.

Considering that too many parameters of the model will bring unnecessary computing overhead, the Inception block is used to replace the original convolution, which reduces the computing overhead while increasing the depth and width of the network, and improves the expressiveness of the model. The specific steps are as follows:

1. The Inception block includes two feature extraction layers, the first layer includes three convolution blocks (denoted as convblock1‐1, convblock1‐2, and convblock1‐3) and a maximum pooling, where convblock1‐1 consists of 64 convolution kernels of size 1*1*1, convblock1‐2 consists of 32 convolution kernels of size 1*1*1, convblock1‐3 consists of 16 sizes of 3*3*3 convolution kernels. The second layer includes three convolution blocks (denoted as c onvblock2‐1, convblock2‐2, and convblock2‐3), where convblock2‐1 consists of 128 convolution kernels of size 3*3*3, convblock2‐2 consists of 32 convolution kernels with a size of 5*5*5, and convblock2‐3 is composed of 32 convolution kernels with a size of 1*1*1. The feature extraction results from convblock1‐1, convblock2‐1, convblock2‐2, and convblock2‐3 are concatenated and fused as the final output of the Inception block.

## 4 RESULTS

### 4.1 Experimental data

**FIGURE 4:**
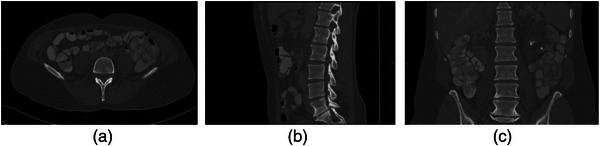
Example of computed tomography (CT) image of lumbar spine. (a) horizontal plane; (b) sagittal plane; (c) coronal plane.

The VerSe 2020 public dataset and our own hospital dataset are used in this paper. Our study is specifically focused on the segmentation of lumbar vertebra. We have selected the CT data which covers the lumbar vertebra. For the VerSe 2020 dataset, after the careful selection, we have obtained a total of 156 CT samples with the lumbar vertebra. The experiments were performed with a five‐fold cross‐validation. For our own hospital dataset, there are 500 samples of CT data in total. All these datasets are provided with ground truth segmentation results from radiologists. A total of 400 samples are utilized for training and other 100 samples are utilized for testing. Figure 4 shows three different sections of a sample of 3D CT image in the dataset, horizontal, sagittal, and coronal.

**FIGURE 5:**
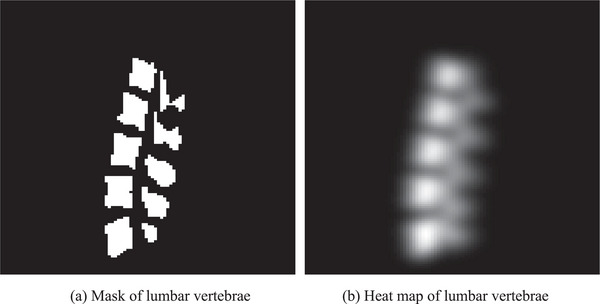
Mask and heat map of lumbar vertebrae for an example.

**FIGURE 6:**
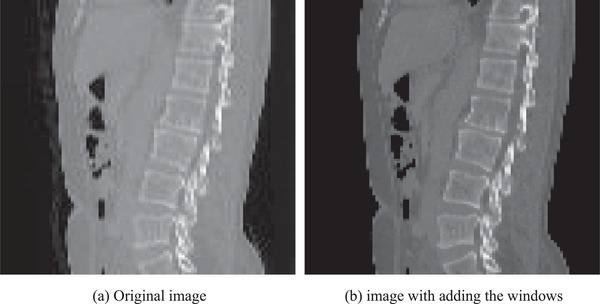
The images before and after adding the windows for an example.

Data preprocessing mainly includes generating spine heatmaps and adding windows in the images. Figure 5 shows an example of the heatmaps and Figure 6 shows the images before and after adding the windows. The lumbar spine heatmap can be obtained by Gaussian smoothing on the lumbar spine mask. This heatmap can be used as a target for a subsequent localization network, which can roughly represent the location of the lumbar spine. Looking at the literature, it can be known that the window and window width of the lumbar skeletal tissue are 400 and 1800, respectively, which can better present the lumbar vertebrae to the network.

### 4.2 Experimental environment

This article uses the python programming language, which integrates the deep learning framework Tensorflow and the image processing library. The code of this article is completed under Windows system using Pycharm as an integrated development environment. The convolutional neural network part uses the popular deep learning framework Tensorflow. The software development environment and hardware equipment used in this paper are as follows.

Software development environment: Windows 10 system, Pycharm 2020.3 × 64 version, Tensorflow 1.16. Hardware equipment: Nvidia 1080T i GPU video memory 1 1G, memory 6 4G, hard disk 2 T.

### 4.3 Evaluation metrics

The performance is evaluated using several commonly utilized evaluation metrics, including dice similarity coefficient (DSC), false negative dice (FND), false positive dice (FPD), maximum symmetric surface distance (MSD), and average symmetric surface distance (ASD). DSC, FND, and FPD are defined by the following equations:

(3) $$DSC=\frac{2*TP}{2*TP+FN+FP}$$

(4) $$FND=\frac{2*FN}{2*TP+FN+FP}$$

(5) $$FPD=\frac{2*FP}{2*TP+FN+FP}\;$$

where TP, FN, and FP represents the true positive voxels, false negative voxels and false positive voxels. DSC can capture the relative overlap of segmentation volumes and ground truth volumes. FND and FPD describes the rate of under‐segmentation and over‐segmentation, respectively. Moreover, DSC, FND, and FPD values range from 0 to 1.

The metrics of MSD and ASD can quantify the distance between segmentation contour surface and ground truth contour surface. MSD is defined by the following equation:

(6) $$\begin{array}{ccc} MSD\; & = & \;\left(max_{i\in seg}\left(min_{i\in gt}\left(d\left(i,j\right)\right)\right)\right.,\\ & & \left.\;max_{j\in gt}\left(min_{i\in seg}\left(d\left(i,j\right)\right)\right)\right)\; \end{array}$$

where d(i,j) represents the contour distance between segmentation results and ground truth. ASD is defined by the following equation:

(7) $$ASD\;=\;mean\left(\left\{B_{seg},B_{gt}\right\}\right)\;$$

(8) $$\begin{array}{ccc} B_{seg} & = & \left(\forall p_{1}\in A_{seg},\;clost\_distance\left(p_{1},p_{2}\right)\right)\\ & & |\exists p_{2}\in \;A_{gt}\; \end{array}$$

### 4.4 Experimental results

**FIGURE 7:**
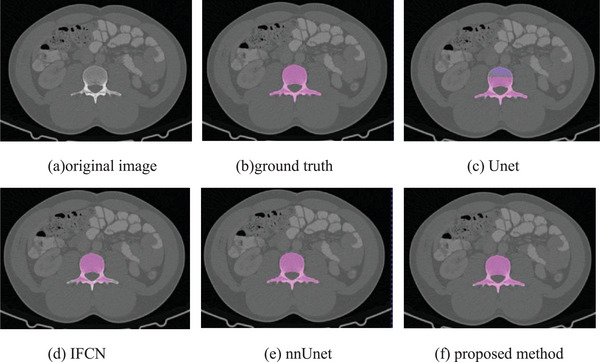
Horizontal visualization of lumbar vertebrae segmentation results of (a) original image, (b) ground truth, (c) Unet, (d) IFCN, (e) nnUnet, and (f) proposed method.

**FIGURE 8:**
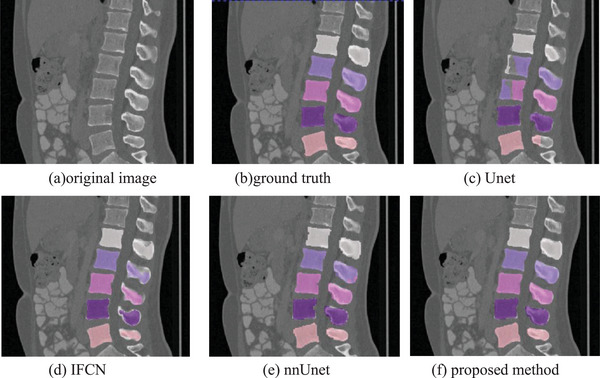
Sagittal visualization of lumbar vertebrae segmentation results of (a) original image, (b) ground truth, (c) Unet, (d) IFCN, (e) nnUnet, and (f) proposed method.

**FIGURE 9:**
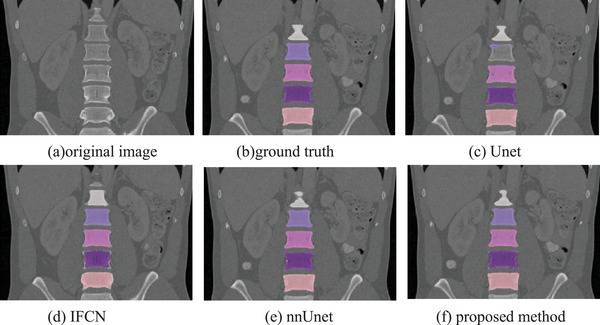
Coronal visualization of lumbar vertebrae segmentation results of (a) original image,(b) ground truth, (c) Unet, (d) IFCN, (e) nnUnet, and (f) proposed method.

To verify the performance of our method, our proposed method is compared with the classic Unet method, the iterative fully convolutional neural method (IFCN)^22^ and the nnUnet method.^25^ In this paper, these methods are experimented on the same dataset as our method, and the same preprocessing method is adopted. Figures 7, 8, and 9 are the different 2D views of the visual representation of the 3D results. From left to right are (a) original image, (b) ground truth, (c) Unet, (d) IFCN, (e) nnUnet, and (f) proposed method. The five lumbar vertebrae are marked with different colors to distinguish them. As can be seen from the figure, the method proposed in this paper can segment the lumbar vertebrae well, which is better than the classic other methods. The proposed method has achieved comparable performance with the promising nnUnet approach.

**FIGURE 10:**
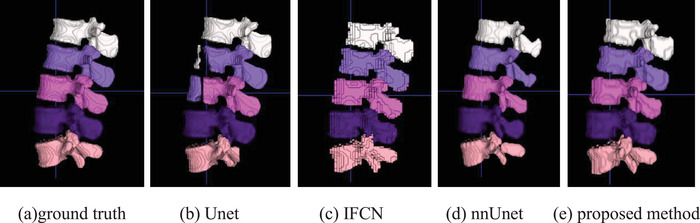
3D visualization of the lumbar vertebrae of (a) ground truth, (b) Unet result, (c) IFCN, (d) nnUnet, and (e) proposed method.

This paper further visualizes the surface of the 3D vertebrae. Figure 10 shows the visualization results of the 3D surface of the segmented vertebrae. From left to right are (a) ground truth, (b) UNet segmentation result, (c) IFCN segmentation result, (e) nnUnet segmentation result, and (f) the segmentation results of this method. The 3D visualization results further verify the superiority of the method. The 3D visualization results are consistent with the 2D results.

We further verified the performance of the proposed method through quantitative analysis on the VerSe dataset and our hospital dataset. The quantitative analysis results of different segmentation results are shown in Tables 1 and 2. The method proposed in our study can achieve better segmentation results. The quantitative analysis is consistent with the visual evaluation. The performance on the hospital dataset is better than the VerSe dataset. The reason is that there are more training data in hospital dataset.

**TABLE 1:** Quantitative evaluation of different methods on the Verse 2020 datasets.

Lumbar segments	Methods	DSC	FND	FPD	MSD	ASD
Lumbar segment 1	UNet	0.8634	0.1943	0.0788	15.73	2.353
	IFCN	0.8814	0.1112	0.1248	7.636	1.890
	nnUNet	0.9067	0.1060	0.0716	6.589	1.872
	Proposed	**0.9125**	**0.0974**	**0.0688**	**6.321**	**1.719**
Lumbar segment 2	UNet	0.8173	0.2768	0.0885	19.14	2.380
	IFCN	0.8826	0.1125	0.1239	15.74	2.002
	nnUNet	0.9090	0.0998	**0.0734**	7.586	1.914
	Proposed	**0.9088**	**0.0968**	0.0856	**6.570**	**1.901**
Lumbar segment 3	UNet	0.7855	0.3507	0.0782	20.81	2.134
	IFCN	0.8834	0.1104	0.1228	11.33	1.938
	nnUNet	0.8930	**0.0897**	0.0778	10.96	1.656
	Proposed	**0.9107**	0.1072	**0.0714**	**6.566**	**1.169**
Lumbar segment 4	UNet	0.8267	0.3277	0.3277	25.46	3.675
	IFCN	0.8802	0.1159	0.0953	17.18	2.569
	nnUNet	0.8813	**0.0953**	0.1214	**10.11**	1.803
	Proposed	**0.9011**	0.0975	**0.0975**	10.85	**1.775**
Lumbar segment 5	UNet	0.8320	0.3262	0.1235	19.10	4.391
	IFCN	0.8481	0.1455	0.0970	12.07	2.352
	nnUNet	0.8790	0.1379	**0.0721**	9.286	0.9870
	Proposed	**0.8878**	**0.1166**	0.0788	**8.762**	**0.9991**

Abbreviations: ASD, average symmetric surface distance; DSC, dice similarity coefficient; FND, false negative dice; FPD, false positive dice; IFCN, iterative fully convolutional neural; MSD, maximum symmetric surface distance.

**TABLE 2:** Quantitative evaluation of different methods on the hospital dataset.

Lumbar segments	Methods	DSC	FND	FPD	MSD	ASD
L1	UNet	0.9470	0.0394	0.0435	6.681	0.3950
	IFCN	0.9612	0.0310	0.0289	5.087	0.2802
	nnUNet	0.9717	0.0298	0.0266	2.828	0.1881
	Proposed	**0.9805**	**0.0206**	**0.0182**	**2.349**	**0.1683**
L2	UNet	0.9511	0.0322	0.0313	5.318	0.2886
	IFCN	0.9661	0.0284	0.0252	3.731	0.2404
	nnUNet	0.9752	0.0255	0.0230	3.162	0.1757
	Proposed	**0.9821**	**0.0200**	**0.0157**	**2.625**	**0.1516**
L 3	UNet	0.9512	0.0380	0.0324	5.385	0.2815
	IFCN	0.9691	0.0276	0.0257	3.599	0.2304
	nnUNet	0.9721	0.0241	0.0214	2.671	0.1865
	Proposed	**0.9802**	**0.0204**	**0.0190**	**2.336**	0.1649
L 4	UNet	0.9499	0.0392	0.0372	6.403	0.2937
	IFCN	0.9668	0.0302	0.0297	3.605	0.2187
	nnUNet	0.9736	0.0262	0.0265	2.975	0.1613
	Proposed	**0.9775**	**0.0229**	**0.0237**	**2.449**	**0.1584**
L 5	UNet	0.9385	0.0792	0.0462	6.881	0.3814
	IFCN	0.9577	0.0689	0.0256	5.174	0.2649
	nnUNet	**0.9730**	**0.0578**	**0.0159**	**3.312**	**0.1979**
	Proposed	0.9700	0.0592	0.0196	3.793	0.2260

Abbreviations: ASD, average symmetric surface distance; DSC, dice similarity coefficient; FND, false negative dice; FPD, false positive dice; IFCN, iterative fully convolutional neural; MSD, maximum symmetric surface distance.

The position part and the inception part is further evaluated with the ablation experiments. Table 3 shows the quantitative analysis of the ablation experiments. Removing one part can slightly decrease the performance of the proposed method. The experiments further evaluate the performance of the developed modules.

**TABLE 3:** Quantitative evaluation of the ablation experiments on the Verse 2020 datasets.

Lumbar segments	Ablation part	DSC	FND	FPD	MSD	ASD
Lumbar segment 1	w/o position	0.8900	0.1072	0.0826	11.072	3.161
	w/o inception	0.8947	0.1063	0.0942	9.282	4.738
	Proposed	**0.9125**	**0.0974**	**0.0688**	**6.321**	**1.719**
Lumbar segment2	w/o position	0.8955	0.1068	0.1021	8.512	2.835
	w/o inception	0.8843	0.1217	0.1095	9.018	3.544
	Proposed	**0.9088**	**0.0968**	**0.0856**	**6.570**	**1.901**
Lumbar segment 3	w/o position	0.8924	0.1303	0.0847	8.060	1.810
	w/o inception	0.8893	0.1322	0.0891	10.11	2.658
	Proposed	**0.9107**	**0.1072**	**0.0714**	**6.566**	**1.169**
Lumbar segment 4	w/o position	0.8826	0.1080	0.1080	15.50	2.451
	w/o inception	0.8716	0.1215	0.1215	18.81	3.597
	Proposed	**0.9011**	**0.0975**	**0.0975**	**10.85**	**1.775**
Lumbar segment 5	w/o position	0.8695	0.1340	0.0970	13.94	3.172
	w/o inception	0.8760	0.1377	0.1003	11.99	2.317
	Proposed	**0.8878**	**0.1166**	**0.0788**	**8.762**	**0.9991**

Abbreviations: ASD, average symmetric surface distance; DSC, dice similarity coefficient; FND, false negative dice; FPD, false positive dice; MSD, maximum symmetric surface distance.

## 5 SUMMARY

Aiming at the difficulties of lumbar vertebrae segmentation in CT images, we propose an automatic lumbar vertebrae segmentation method based on deep learning. The method mainly includes two parts: lumbar vertebra positioning and lumbar vertebrae segmentation. First of all, we propose a lumbar spine localization network of Unet network, which can directly locate the lumbar spine part in the image. Then, we propose a three‐dimensional XUnet lumbar vertebrae segmentation method to achieve automatic lumbar vertebrae segmentation. The method proposed in this paper is validated on the lumbar spine CT images on the public dataset VerSe 2020 and our hospital dataset. Through qualitative comparison and quantitative analysis, the experimental results show that the method proposed in this paper can obtain good lumbar vertebrae segmentation performance, which can be further applied to detection of spinal anomalies and surgical treatment.

## AUTHOR CONTRIBUTIONS

Hongjiang Lu and Mingying Li: methodology, investigation, formal analysis, writing ‐ original draft. Kun Yu and Yingjiao Zhang: data curation, investigation, formal analysis, validation. Liang Yu: conceptualization, supervision, writing ‐ review & editing.

## CONFLICT OF INTEREST STATEMENT

The authors declare no conflicts of interest.

## Data Availability

All data included in this study are available upon request by contact with the corresponding author.
